# Structure‐dependent effects of amyloid‐β on long‐term memory in *Lymnaea stagnalis*

**DOI:** 10.1002/1873-3468.12633

**Published:** 2017-04-24

**Authors:** Lenzie Ford, Michael Crossley, Devkee M. Vadukul, György Kemenes, Louise C. Serpell

**Affiliations:** ^1^ Sussex NeuroscienceSchool of Life SciencesUniversity of SussexBrightonUK; ^2^ Present address: Department of NeuroscienceColumbia UniversityNew YorkNY10032USA; ^3^ Present address: Howard Hughes Medical InstituteColumbia UniversityNew YorkNY10032USA

**Keywords:** amyloid beta, classical conditioning, long‐term memory, *Lymnaea*, oligomer

## Abstract

Amyloid‐β (Aβ) peptides are implicated in the causation of memory loss, neuronal impairment, and neurodegeneration in Alzheimer's disease. Our recent work revealed that Aβ 1–42 and Aβ 25–35 inhibit long‐term memory (LTM) recall in *Lymnaea stagnalis* (pond snail) in the absence of cell death. Here, we report the characterization of the active species prepared under different conditions, describe which Aβ species is present in brain tissue during the behavioral recall time point and relate the sequence and structure of the oligomeric species to the resulting neuronal properties and effect on LTM. Our results suggest that oligomers are the key toxic Aβ1–42 structures, which likely affect LTM through synaptic plasticity pathways, and that Aβ 1–42 and Aβ 25–35 cannot be used as interchangeable peptides.

## Abbreviations

**Aβ**, amyloid‐β

**AD**, Alzheimer's disease

**APP**, amyloid precursor protein

**CGC**, cerebral giant cell

**HFIP**, hexafluoroisopropanol

**LTM**, long‐term memory

**SEM**, standard error mean

**TEM**, transmission electron microscopy

Amyloid β (Aβ) is cleaved from the amyloid precursor protein (APP) to produce a range of Aβ isoforms of which Aβ 1–40 and Aβ 1–42 are the most common. This APP cleavage process is well‐defined, with Aβ peptides predominantly being produced *via* ɑ‐ or β‐ and ɣ‐secretases 1, 2. Alongside Aβ peptides, other APP fragments are produced in the cleavage process and are believed to play neuroprotective and neurotrophic roles in the brain 1, 2. The function of APP and its peptides are unknown, but appear to have an important role in neuromuscular junction formation, synaptic transmission, and ion channel function 3. In Alzheimer's disease (AD), the shift from healthy protective function to pathogenic cell death arises from an increase in Aβ 1–42 production and oligomerization 1, 4, 5. Other Aβ peptides of various lengths have also been found in both AD brains and cerebrospinal fluid 6, 7, being produced *via* caspases and proteolytic degrading enzymes 1. These fragments have been suggested to play an important role in pathology 1.

One of these shorter fragments, Aβ 25–35, has been detected in AD plaques and is a known cleavage product of Aβ 1–40 racemized at d‐Ser26 8. Aβ 25–35 may represent the toxic core of the more physiologically prevalent toxic species Aβ 1–42 9, and it displays similar fibrillization through β sheet formation 10. For this reason, many labs utilize Aβ 25–35 as a cost‐effective means of studying Aβ. Of the different length peptides, available both synthetically and in AD‐related tissues, it is generally agreed that these peptides are toxic when they exist as small, prefibrillar oligomers 11, 12, 13, 14, 15. Specifically, dimers and dodecamers have been directly linked to toxicity and behavioral disruptions 12, 13, 16, 17, 18, although others suggest that all soluble low‐n oligomers could be toxic 19, 20. These toxic oligomers produce AD pathology by first disrupting synapse function in memory‐encoded neuronal circuitry, further developing into synaptic degeneration, and finally full cell death 12, 13, 21.

Amyloid precursor protein is highly evolutionarily conserved, with > 95% sequence homology existing across mammalian species and high homology within invertebrate species 22, 23. Many invertebrate model organisms have been used for Aβ and AD studies 24, 25. For example, *Drosophila* has an APP ortholog, APPL 26, an ɑ‐secretase ortholog 27, and components of ɣ‐secretase 28, 29, 30. This ɣ‐secretase can process human APP 31, 32 and human APP can be cleaved to produce Aβ in flies, suggesting an endogenous β‐secretase‐like protease in *Drosophila* 32. APP and the protease processing system (presenilin 1 and 2) are well conserved across the animal kingdom and APP mRNA expression has been shown in the ganglia of *Apylsia californica* 33, which is closely related to *Lymnaea*.

Aβ and AD research has only rarely branched into molluscan model systems, although these offer a wealth of information on cellular and molecular mechanisms of memory function and dysfunction by providing uniquely tractable models in the field 34. Indeed, the use of mollusks such as the sea slug *A. californica* and the pond snail *Lymnaea stagnalis* helped build much of the molecular and electrophysiological understanding of learning and memory 34, 35, 36, 37, 38, 39, 40. The first group to utilize a mollusk in Aβ memory studies considered Aβ 25–35 in the land snail *Helix lucorum* 41. In these experiments, the researchers reported that the animals’ conditioned food aversion reflex was inhibited when Aβ was administered before training 41. Our lab expanded the Aβ studies in mollusks, finding that Aβ 1–42 and Aβ 25–35 disrupted consolidated long‐term memory (LTM) in the pond snail *L. stagnalis* 42.

Our studies brought about a very intriguing question: Are the two peptides affecting memory consolidation *via* similar pathways? Although both peptides ultimately disrupted consolidated long‐term memory prior to neuronal death, there were significant differences in: (a) peptide production, (b) morphology, (c) quantity of oligomers in the hemolymph, and (d) effects on neuron electrical properties.

1. Firstly, although Aβ 1–42 and Aβ 25–35 both disrupted consolidated long‐term memory, the two peptides were produced under very different conditions 42. Aβ 1–42 was administered at 1 μm directly into the hemolymph, with an expected final concentration of 1 nm. Moreover, Aβ 1–42 was solvent prepared. In this preparation method, the lyophilized peptide is solubilized in a fluorinated alcohol for disaggregation, dried, and resolubilized in DMSO. The solubilized peptide then undergoes removal of solvent *via* a desalting column and buffer‐exchange into a final normal saline solution, and is centrifuged to remove any insoluble aggregates. This solvent preparation method (see Materials and methods) has been well studied 42, 43, 44 and produces maximal soluble Aβ 1–42. In contrast, lyophilized Aβ 25–35 was solubilized directly into saline solution (as for *H. lucorum* 41), incubated for 2 h at room temperature, and then administered at 0.1 mm with an expected final concentration of 0.1 μm 42. When injected at 1 μm, memory was no longer disrupted which suggests that a significantly higher concentration of Aβ 25–35 was necessary for similar behavioral effects 42.
2. Comparison of the morphological features of Aβ 1–42 and Aβ 25–35 peptides over a 24‐h *in vitro* assembly by transmission electron microscopy (TEM) revealed significant differences between the structures formed by the two peptides under these conditions. Aβ 1–42 followed a self‐assembly pathway from oligomeric, to protofibrillar, and finally fibrillar states while Aβ 25–35 was predominantly crystalline in morphology and aggregated further over time 42.
3. Hemolymph of both Aβ 1–42‐ and Aβ 25–35‐treated animals underwent formic acid extraction, immunogold labeling with Aβ oligomer antibody Nu1 12, and were visualized with TEM. Both samples contained more Aβ oligomer labeling than buffer‐treated controls, but with a 600‐fold more labeling in Aβ 1–42‐treated animals compared to Aβ 25–35 42.
4. Finally, the two peptides disrupted properties of the *Lymnaea* nervous system differently. Aβ 25–35 caused a decrease in input resistance and an abolition of the learning‐induced depolarization of the membrane potential of the cerebral giant cell (CGC), a key neuron underlying memory 45, 46, while Aβ 1–42 had no detectable effect on CGC electrical properties 42.

These findings led us to consider whether the observed behavioral and electrophysiological differences in the Aβ peptides are due to the different lengths and sequences of the peptides, or to the structure the peptides adopted by the 24‐h postinjection time point when the memory test was conducted. If the primary structure is the critical difference between the two peptides, the method of preparation should not alter the peptide's effect on behavior or electrophysiology. However, if the peptide's effect on behavior is related to its structure at the 24‐h postinjection time point, then a difference in peptide preparation should have drastic effects on the resulting behavior and electrophysiology. To address these questions, we prepared Aβ 25–35 using the previously mentioned solvent preparation method 44, and report the resulting changes in LTM, electrical neuronal properties, peptide morphology, and quantity of oligomers in the hemolymph after 24 h of *in vivo* incubation. Here, we reveal significant differences in the effects of peptides formed under different conditions and with different structures; expanding knowledge of the effects of oligomeric Aβ on memory and cellular functions in the brain.

## Materials and methods

### Experimental animals

Pond snails, *L. stagnalis*, were bred and maintained in 18–22 °C copper‐free water in large holding tanks, with a 12 : 12 h light–dark cycle. The animals were fed twice a week with Tetra‐Phyll (TETRA Werke, Melle, Germany) and with lettuce three times a week. Three days before each experiment an appropriate number of animals were transferred into the behavioral laboratory where they were kept in smaller tanks in a food‐deprived state before the experiments commenced.

### Preparation and systemic application of Aβ peptides

Aβ peptides were solvent prepared, as described previously 42, 44. Briefly, 0.2 mg Aβ Fragment 25–35 (Sigma‐Aldrich, Irvine, UK) or Aβ 1–42 (rPeptide; Bogard, GA, USA) were solubilized in hexafluoroisopropanol (HFIP; Sigma‐Aldrich) to disaggregate the peptides, and then dried completely to remove HFIP. This protocol has been optimized 43 and has been shown to reproducibly produce soluble, oligomeric Aβ 1–42 42, 44, 47. Once HFIP was completely evaporated, dry DMSO (Sigma‐Aldrich) was added to the Aβ. The Aβ was then added to a prepared Zeba buffer‐exchange column (ThermoFisher Scientific, Paisley, UK) with a normal saline solution (50 mm NaCl, 1.6 mm KCl, 2 mm MgCl_2_. 6H_2_O, 3.5 mm CaCl_2_ × 2H_2_O, 10 mm HEPES [4‐(2‐hydroxyethyl)‐1‐piperazine‐ ethanesulfonic acid], pH 7.9) 48 stack and centrifuged for 30 min at 16 000 ***g***, 4 °C to remove insoluble structures and all remaining solvents 49. This final step is critical for removing fibrillar species, leaving only soluble, oligomeric Aβ 44. Protein concentration was calculated by measuring optical density at 280 nm using a NanoDrop spectrophotometer (Thermo Fisher, Paisley, UK) and correcting for the molar absorption coefficient of each peptide. Aβ peptides were then diluted to the 1 μm working concentration in 100 μL using normal saline solution at 20 °C, and were systemically injected into the animals directly after preparation using a 1‐mL syringe with 30‐gauge precision glide needles (Becton Dickinson, Oxford, UK). For vehicle control animals, 100 μL of normal saline solution was injected.

### Formic acid‐extracted hemolymph preparation

After 24 h *in vivo* incubation of solvent‐prepared Aβ 25–35, roughly 1 mL of hemolymph was extracted from each snail and submitted to formic acid extraction, as described previously 42, 50. Briefly, the hemolymph was mixed with equal volumes 0.4% diethylamine/100 mm NaCl. About 400 μL was then centrifuged at 16 000 ***g*** for 1 h, 4 °C. Supernatant was aspirated and 200 μL 1 m Tris pH 7.4 was added to the pellet. Four hundred microliters of cold formic acid was added and the sample was sonicated and then centrifuged at 16 000 ***g*** for 1 h, 4 °C. The supernatant was neutralized in 4 mL 1 m Tris, 0.5 m Na_2_HP0_4_, which was again centrifuged at 16 000 ***g*** for 1 h, 4 °C. The supernatant was neutralized with 1/10 volume 1 m Tris, pH 6.8. The samples were stored at −80 °C until used for imaging by TEM.

### Transmission Electron Microscopy

As previously described 42, 4 μL of the formic acid‐extracted hemolymph sample was pipetted on to Formvar/carbon coated 400‐mesh copper grids (Agar Scientific, Essex, UK), washed with Milli‐Q water (EMD Millipore, Watson, UK), and negative stained with 2% uranyl acetate for 1 min. Grids were allowed to air dry. After initial imaging the samples were immunogold labeled with 1 μg·mL^−1^ Nu1 (Klein Laboratory) 12, a mouse conformational antibody raised against oligomeric Aβ, and then labeled with goat anti‐mouse 10 nm gold‐conjugated secondary antibody (BBI Solutions OEM Ltd., Cardiff, UK) to label oligomeric structures. All grids were examined in a Hitachi 7100 TEM at 100 kV and digital images acquired with an axially mounted (2K × 2K pixel) Gatan Ultrascan 1000 CCD camera (Gatan UK, Oxford, UK).

Negative staining of solvent‐prepared Aβ 25–35 was used to monitor peptide morphology over the incubation time. Aliquots of 100 μm Aβ 25–35 were allowed to incubate in normal saline solution (50 mm NaCl, 1.6 mm KCl, 2 mm MgCl_2_ × 6H_2_O, 3.5 mm CaCl_2_ × 2H_2_O, 10 mm HEPES, pH 7.9 at 20 °C) in a closed Eppendorf tube for 0, 3, or 24 h. This *in vitro* incubation method previously produced reliable and reproducible results for buffer‐prepared Aβ 25–35 and solvent‐prepared Aβ 1–42 42. Samples were prepared and images acquired as stated above. This experiment was conducted three times, to ensure assembly was consistent.

### Single‐trial food‐reward classical (CS+US) conditioning

Using well‐established methods 45, *L. stagnalis* underwent single‐trial food‐reward classical conditioning in which the conditioned stimulus (amyl acetate) and the unconditioned stimulus (sucrose) were paired. An unpaired control was not used, as naive controls show no difference from unpaired controls behaviorally 51, 52 or electrophysiologically 53. Both the vehicle‐injected control and Aβ‐injected groups were trained. The naïve groups were not trained and were not injected, but underwent the same food‐deprivation/feeding schedule and handling as the experimental groups.

### Electrophysiology

The two‐electrode current‐clamp‐based electrophysiology method employed to test the electrical properties of the CGCs has been described in detail elsewhere 45. Briefly, the cerebral ganglia (location of the CGCs) were desheathed and treated with a solid protease (Sigma type XIV; Sigma‐Aldrich) to soften the inner sheath for intracellular recording. Sharp electrodes (5–20 MΩ) were filled with 4 m potassium acetate. Signals from the intracellular electrodes were amplified using Axoclamp 2B (Axon Instrument, Molecular Devices, Sunnyvale, CA, USA) and NL 102 (Digitimer, Hertfordshire, UK) amplifiers and digitized at 2 kHz using a micro 1401 Mk II interface and analyzed using spike 2 software (version 5.21; Cambridge Electronics Design, Cambridge, UK). The CGC membrane potential and input resistance as well as action potential characteristics (frequency, amplitude, half‐width, and after‐hyperpolarization amplitude) were analyzed over a 100‐s period recorded 120 s after the initial electrode impalement. This is sufficient time to allow the cell to recover from impalement 45, 53, 54.

### Statistical analysis

Data were analyzed using graphpad prism software (version 4.03; GraphPad Software Inc., San Diego, CA, USA). Normality was tested using D'Agostino and Pearson omnibus normality test. Data were first analyzed with one‐way ANOVA followed by Tukey's multiple comparison to establish significance (criterion, *P* < 0.05).

## Results

### Solvent‐prepared Aβ 25–35 has no significant effect on *Lymnaea* LTM recall or electrical neuronal properties

Two important questions in the Aβ and AD field still demand elucidation: (a) Can synthetic Aβ 25–35 reliably be used in place of synthetic Aβ 1–42?; (b) Are the observed behavioral effects of various Aβ peptides on consolidated long‐term memory due to different primary structures, or due to the final conformation of these peptides? We speculated briefly about the answers to these questions previously after we discovered that both Aβ 1–42 and Aβ 25–35 disrupted consolidated LTM in *Lymnaea* at different concentrations and potentially *via* different pathways 42. Here, we aim to directly address each question by comparing synthetic peptide preparation methods and observing the resulting effect on behavior. For solvent‐prepared Aβ, peptides are solubilized in HFIP and undergo column purification and centrifugation to remove aggregated species. This method produces soluble Aβ 42, 44, 47, which is then diluted into normal saline. Previously, buffer‐prepared Aβ peptides had been solubilized in normal saline and vortexed briefly 41, 42.

**Figure 1:**
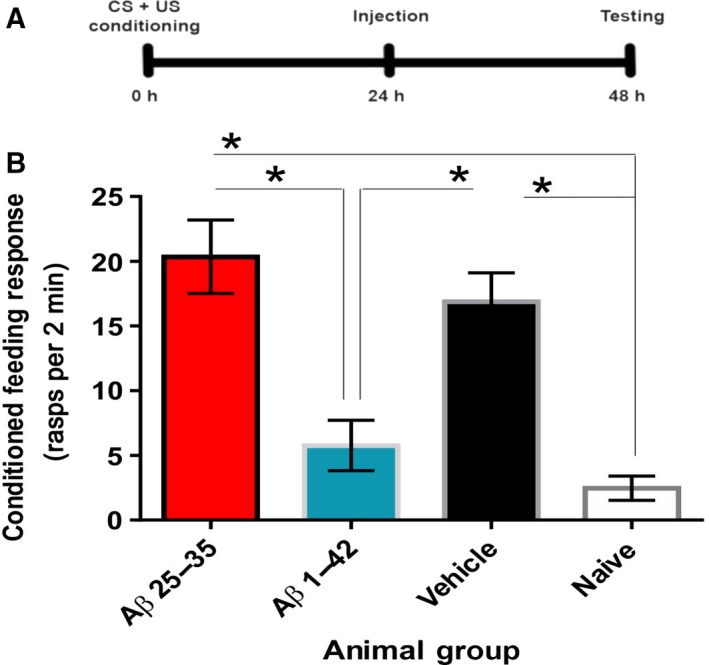
Solvent‐prepared Aβ 25–35 (1 μm) does not cause memory impairment when allowed to incubate *in vivo* for 24 h. (A) Timeline of the experiment. CS, conditioned stimulus; US, unconditioned stimulus. (B) Four starved animal groups [solvent prepared Aβ 25–35 (*n* = 23) and Aβ 1–42 (*n* = 13), buffer‐only vehicle (*n* = 18), naïve (untreated and untrained) (*n* = 17)] were tested for rasp rate to amyl acetate, a measure of the feeding response to the conditioned stimulus. Means ± standard error mean (SEM) values are shown. Asterisks indicate behavioral responses that are significantly different between groups. ANOVA,* P* < 0.0001. Tukey's multiple comparisons with *P* < 0.05: Aβ 25–35 vs. Naïve, Aβ 25–35 vs. Aβ 1–42, Vehicle vs. Naïve, Vehicle vs. Aβ 1–42.

To tackle these questions, we used a single‐injection and single‐trial behavioral paradigm in a tractable animal model of long‐term memory. *Lymnaea stagnalis* were trained using single‐trial food‐reward classical conditioning 45, injected with 1 μm solvent‐prepared Aβ 25–35 or Aβ 1–42 24 h post‐training, and tested 48 h post‐training (Fig. 1A). Aβ‐treated animals were compared to vehicle‐treated control animals and naïve animals, shown in Fig. 1. Aβ 25–35 (1 μm) did not cause behavioral deficits; instead, the animals in Aβ 25–35 (1 μm) group exhibited similar behavioral responses to vehicle‐injected control animals. Both Aβ 25–35 (1 μm)‐treated and vehicle‐injected animals exhibited a significantly greater feeding response to the conditioned stimulus compared to naive and Aβ 1–42 (1 μm)‐treated animals (Fig. 1B). Thus, solvent‐prepared Aβ 25–35 (1 μm) does not disrupt memory in contrast to solvent‐prepared Aβ 1–42 (1 μm), when applied at equal concentrations.

**Figure 2:**
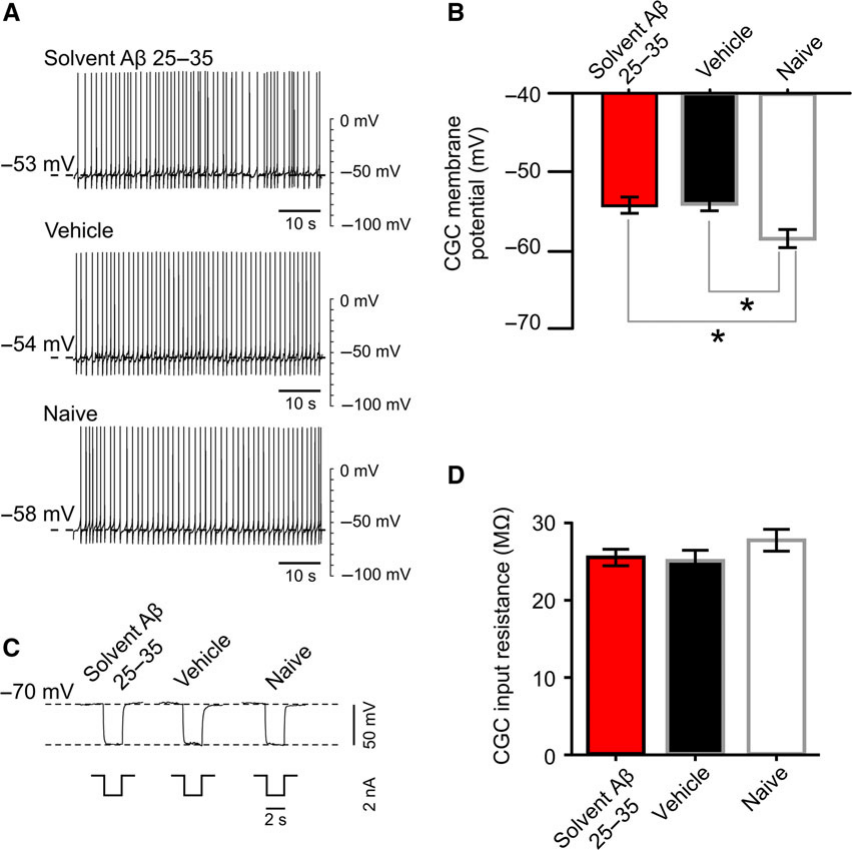
Electrophysiological effects of solvent‐prepared Aβ 25–35 treatment. (A) Examples of electrophysiological recordings of CGC membrane potential and tonic firing activity under control and experimental conditions. (B) Membrane potential, represented graphically [vehicle/buffer control (*n* = 12); Aβ 25–35 (*n* = 10); naïve (*n* = 12)]. Means ± SEM values are shown. One‐way ANOVA,* P* = 0.0052. Tukey's tests with *P* < 0.05: Vehicle vs. Naïve, Aβ 25–35 vs. Naïve (indicated by asterisks). (C) Examples of electrophysiological recordings of CGC membrane resistance under control and experimental conditions. (D) Membrane resistance, represented graphically [vehicle/buffer control (*n* = 11); Aβ 25–35 (*n* = 10); naïve (*n* = 11)]. Means ± SEM values are shown. One‐way ANOVA,* P* = 0.3217.

We continued our investigation to determine whether solvent‐prepared Aβ 25–35 could alter spike characteristics and two of the key electrical properties of the CGCs, membrane potential and membrane resistance, both of which were shown to be affected by buffer‐prepared Aβ 25–35 42. Of the measured parameters, learning‐induced depolarization of the CGC soma membrane was linked to long‐term memory in previous studies 46 with the other parameters remaining unaffected by single‐trial classical conditioning 45. The hypothesis we were testing here was that the solvent‐prepared Aβ 25–35's inability to disrupt memory was predominantly due to a lack of effect on the CGC's membrane potential. The other parameters were measured because it could not be ruled out that similar to its buffer‐prepared version, solvent‐prepared Aβ 25–35 would abolish learning‐induced depolarization of the CGC soma membrane but the memory impairing effects of this change would be compensated for by homeostatic changes in spike characteristics or input resistance. In accordance with previous findings 45, no change was observed in key parameters of the CGC action potentials, such as spike frequency, amplitude, half‐width, or after‐hyperpolarization after classical conditioning (S1). The input resistance of the CGC soma membrane was also unaffected by solvent‐prepared Aβ 25–35 (Fig. 2). Importantly, the CGC's membrane potential remained depolarized, similar to vehicle controls (Fig. 2).

### Characterization of solvent‐prepared Aβ 25–35

From the combined behavioral and electrophysiological experiments from Figs 1 and 2, we were very curious to understand the conformation and state of oligomerization of the apparently benign, solvent‐prepared Aβ 25–35. We predict that by altering the method of peptide preparation from buffer prepared 41, 42 to solvent prepared, the conformational state of Aβ 25–35 has been altered and this has affected Aβ 25–35's ability to disrupt nonsynaptic plasticity in *Lymnaea* central nervous system and LTM 42, 45. An intriguing point is thus raised: a simple alteration of peptide preparation, and thus conformation of the protein, is directly related to its function. We continued our investigation into this benign Aβ 25–35 to understand what morphological change occurred as a result of solvent preparation.

**Figure 3:**
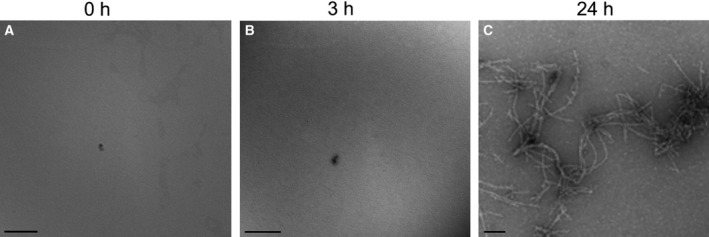
Solvent‐prepared Aβ 25–35 fibrilizes when allowed to incubate in normal saline solution for 24 h. About 100 μm solvent‐prepared Aβ 25–35 was prepared as described in Materials and methods and allowed to aggregate in normal saline solution over a 24‐h period. Samples were taken at 0, 3, and 24 h, negative stained, and imaged using the TEM. The peptide self‐assembles over the 24‐h period. (A) No observable Aβ 25–35 species are found at the 0‐h time point. (B) No observable Aβ 25–35 species are found at the 3‐h time point. (C) Aβ 25–35 fibrils are found at the 24‐h time point. Scale bars represent 100 nm.

Aβ 1–42 self‐assembles from soluble monomer, to soluble low‐n oligomers and large‐n oligomers, and finally to cross‐β fibrils 9. Along this pathway, morphologically distinct oligomeric, protofibrillar, and fibrillar states can be observed using negative stain TEM. To investigate the assembly of the solvent‐prepared Aβ 25–35, the peptide was allowed to assemble *in vitro* at room temperature in a closed Eppendorf tube over a 24‐h period and samples were examined by TEM after 0, 3, and 24 h of incubation. There were no observable species at either the 0‐ or 3‐h time points, suggesting the peptide remains in an unassembled or low‐n oligomeric state. Due to negative stain method constraints, a resolution limit of about 3 nm exists 54. Therefore, it is unlikely that a monomer or low‐n oligomer of Aβ 25–35 structure can be visualized using this method. By 24 h, amyloid‐like fibrils had formed (Fig. 3). These fibrils have a pronounced curved appearance. There was no evidence of higher order oligomer formation at any observed time point (Fig. 3A,B).

**Figure 4:**
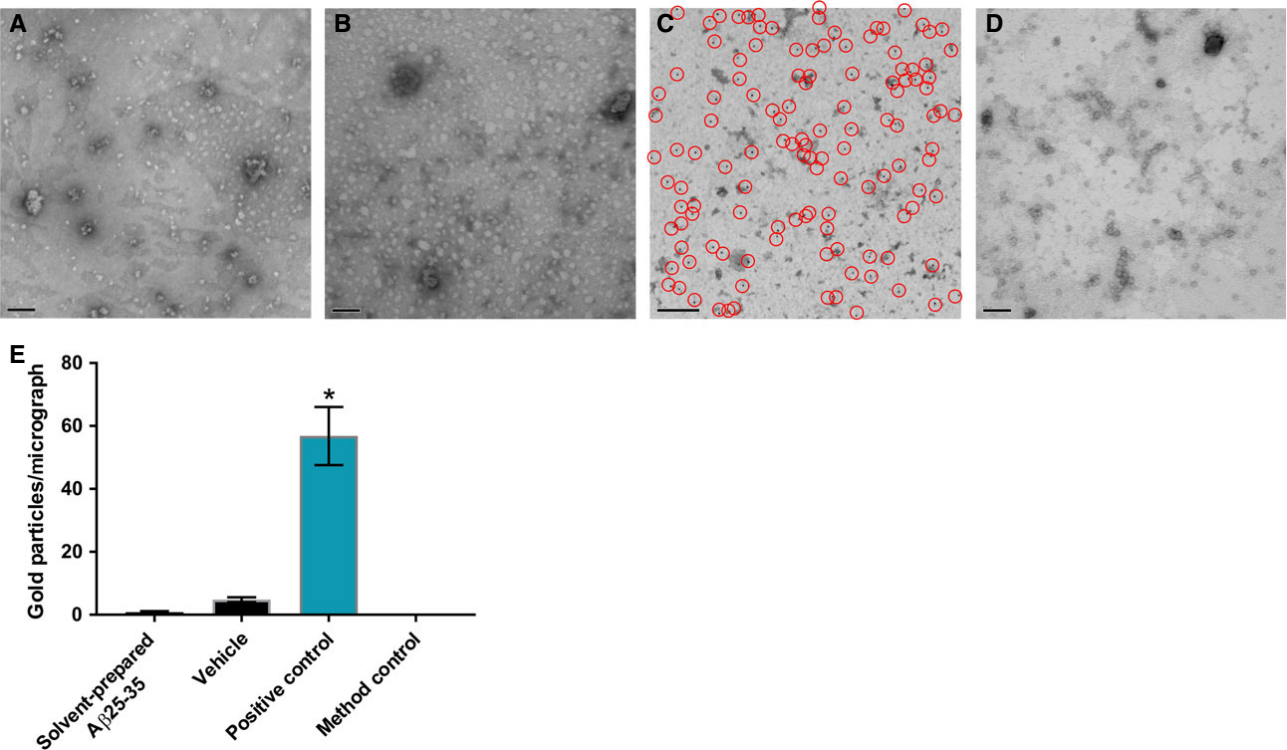
Oligomeric Aβ is not found in the hemolymph of animals after 24 h *in vivo* incubation with solvent‐prepared Aβ 25–35. (A) Micrograph of negative stained and Nu1 immunogold‐labeled, formic acid‐extracted hemolymph from animals treated with 1 μm solvent‐prepared Aβ 25–35 after 24 h *in vivo* incubation. (B) Micrograph of negative‐stained and Nu1 immunogold‐labeled, formic acid‐extracted hemolymph from animals treated with vehicle after 24 h *in vivo* incubation. (C) Positive control: Micrograph of negative stained and Nu1 immunogold‐labeled, formic acid‐extracted hemolymph from animals treated with 1 μm solvent‐prepared Aβ 1–42 after 24 h *in vivo* incubation. Red circles indicate gold particles. (D) Method control: Micrograph of negative stained and secondary antibody‐only labeled, formic acid‐extracted hemolymph from animals treated with 1 μm solvent‐prepared Aβ 1–42 after 24 h *in vivo* incubation. Scale bars in A–D represent 100 nm. (E) Graphical representation of immunogold labels present in micrographs. Aβ 25–35 *n* = 20, Vehicle *n* = 16, positive control *n* = 11, antibody control *n* = 16. Means ± SEM values are shown. Asterisk indicates significant differences in gold particles per image between the positive control and each of the other groups (One‐way ANOVA,* P* < 0.0001; Tukey's, *P* < 0.05).

The resolution limitations of negative stain may be the reason why no oligomeric species were observed. To examine whether oligomeric Aβ 25–35 is found *in vivo* following administration of solvent preparation samples, Aβ 25–35 was extracted from the animals’ hemolymph 24 h after treatment using formic acid and prepared for immunogold labeling and imaging using TEM 50. Soluble fractions were added to a TEM grid, negative stained, immunogold labeled using the anti‐Aβ oligomer antibody Nu1 12 and a gold‐conjugated secondary antibody, and imaged using TEM. Even if the oligomers are too small for visualization by TEM, the antibody gold particles will indicate areas where Aβ 25–35 oligomers are present. Extracts from animals treated with solvent‐prepared Aβ 25–35 expressed negligible labeling, less than 1 gold label per micrograph (Fig. 4A). This was similar to the vehicle‐injected (buffer only) animal oligomer levels (Fig. 4B). Aβ 1–42‐injected positive controls labeled very well with this immunogold‐labeling method (Fig. 4C) and the Nu1 antibody was validated by a lack of labeling in the secondary antibody‐only method control (Fig. 4D). The only sample with significant immunogold labeling was the positive control (Fig. 4). This suggests that nonspecific antibody labeling was very low and that oligomeric Aβ 25–35 species were not present at detectable levels in the sample extracted from treated animals.

## Discussion

Solubilized Aβ peptides are commonly used in amyloid studies. Aβ 1–42 is the predominant toxic species, as it has been linked to AD and the accompanying memory loss and neuronal death 9. However, Aβ 25–35 is still commonly utilized as an affordable alternative to Aβ 1–42, as it has been shown to be toxic to cells and retains similar structural properties 1, 6, 7, 8, 9, 10. Much focus has gone into appropriate preparation of Aβ peptides, and standardization of peptide preparation will likely decrease experimental variability between research groups. This research is critical, as varying the preparation of synthetic peptides is known to result in morphologically and functionally distinct Aβ 50, 55, 56. We intended to study the effect of this variability in Aβ peptide preparation by comparing previously published work on solvent‐prepared Aβ 1–42 and buffer‐prepared Aβ 25–35, with our current work on solvent‐prepared Aβ 25–35. The effect of Aβ preparation on downstream memory mechanisms remains unclear. Here, we considered how Aβ preparation using solvent affects the range of conformational species produced and their effects on a highly tractable model animal. We considered Aβ's effect on *Lymnaea* LTM, a number of electrical properties of a key neuron in *Lymnaea*'s central nervous system, the presence of oligomers in *Lymnaea* hemolymph, and the peptide's morphological change over time *in vitro*. This work not only identifies preparation methods of functionally relevant Aβ 1–42 and Aβ 25–35, but may indicate that care should be taken when replacing Aβ 1–42 with Aβ 25–35. Indeed, the two peptides have drastically differing effects on *Lymnaea* behavior and nonsynaptic plasticity when prepared and applied under memory‐disrupting conditions and concentrations.

We observed here and in previous work that memory recall 24 h postinjection, 48 h post‐training is only disrupted in Aβ‐treated animals that retain oligomeric species in their hemolymph after 24 h *in vivo* incubation 42. This is unsurprising, as other labs have suggested that the solvent used to dissolve synthetic Aβ affects the initial conformation and aggregation kinetics 57. Importantly, the difference in primary structure of Aβ 1–42 and Aβ 25–35 drastically alters how these peptides fold in different environments, as revealed by TEM studies by comparing solvent‐prepared Aβ 1–42 and Aβ 25–35 with buffer‐prepared Aβ 25–35 42. This emphasis on environmental influence is critical to AD research, as Aβ of varying lengths have been identified in the disease 6, 7. The research presented here suggests that the formation of intermediates by Aβ peptides is heavily influenced by preparation method, and when Aβ 25–35 is solvent prepared, it does not form pathological species. Only when prepared to form nonamyloid‐like crystalline structures does Aβ 25–35 have the pathologically relevant effect of impairing LTM 42.

**Figure 5:**
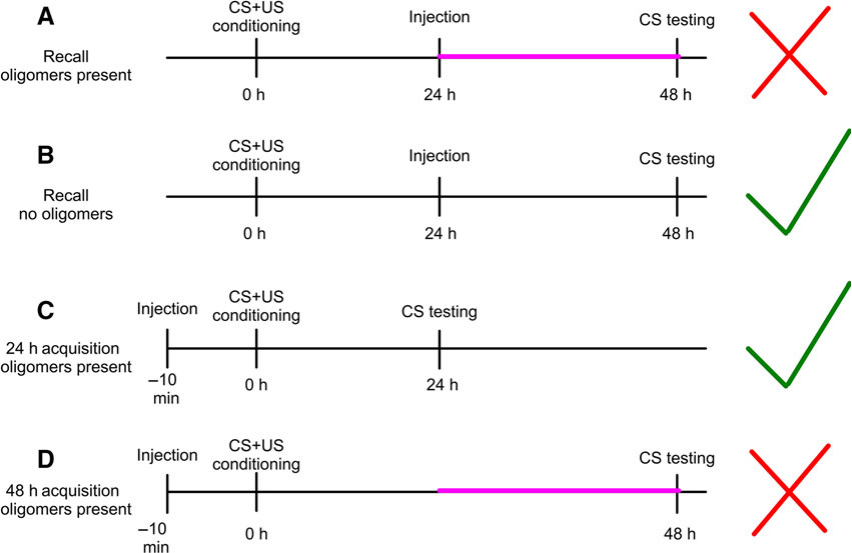
Behavioral memory time lines. (A) 24‐h *in vivo* incubation, memory recall time point with Aβ treatments that result in the presence of *in vivo* oligomers after 24 h. Memory is inhibited. (B) 24‐h *in vivo* incubation, memory recall time point with Aβ treatments that exhibit no *in vivo* oligomers after 24 h. Memory functions correctly. (C) 24‐h *in vivo* incubation, memory acquisition time point with Aβ treatments that result in the presence of *in vivo* oligomers after 24 h. Memory functions correctly. (D) 48‐h *in vivo* incubation, memory acquisition time point with Aβ treatments that result in the presence of *in vivo* oligomers after 24 h. Memory is inhibited. The red ‘×’ indicates experiments where memory is inhibited. The green ‘check’ indicates experiments where memory functions correctly. The pink line indicates the 24–48‐h postconditioning time point that may be vulnerable to Aβ oligomers.

Importantly, only specific phases of memory seem to be vulnerable to Aβ oligomers in these studies. We found that the 24–48‐h postconditioning time point is vulnerable to Aβ oligomers 42. This time point is not vulnerable to Aβ nonoligomeric species 42. The 0–24 h post‐training time point is also not disrupted by Aβ treatment (Fig. 5), regardless of whether oligomeric species are present or not 42. This supports previous studies that showed that Aβ oligomers are the memory‐disrupting species 13, 14, 16, 18 and research that suggests that memory phases dependent upon synapse structure remodeling are vulnerable to Aβ 55, 58, 59. Our work supports the emerging hypothesis that oligomeric Aβ affects memory by altering new synaptic growth or synaptic rearrangement 60 (Fig. 5), which is necessary for the persistence of long‐term memory 61. Our exploration of structure‐dependent effects of Aβ on behavioral function critically enhances the field in a novel way. Due to the nonamyloid‐like crystallization of memory‐disrupting Aβ 25–35, we believe this could be an artifact of improperly folded peptide, which can then disrupt the electrical properties of neurons in a non‐native way. However, further experiments are needed to be certain. This in‐depth focus of Aβ structure, and its influence on neuronal circuitry and memory time points, narrows the scope for future studies investigating molecular memory and drug targeting.

## Author contributions

LF, GK, and LCS conceived the project. LF and LCS designed the imaging experiments, which were performed by LF and DV, and analyzed by LF. LF designed, performed, and analyzed the behavioral experiments. LF and MC designed the electrophysiology experiments, which were performed and analyzed by MC. LF, GK, and LCS wrote the paper and all authors edited the manuscript.

## Data sharing statement

The datasets supporting the conclusions of this article are included within the article and its supporting information.

## Supporting information


**Fig. S1.** Solvent‐prepared Aβ 25–35 does not affect certain intrinsic neuronal properties.
